# Addressing the Urgent Need for Clinical Nutrition Education in PostGraduate Medical Training: New Programs and Credentialing

**DOI:** 10.1016/j.advnut.2024.100321

**Published:** 2024-10-16

**Authors:** Sundar Krishnan, Trevor Sytsma, Paul E Wischmeyer

**Affiliations:** 1 Department of Anesthesiology, Duke University School of Medicine, Durham, United States; 2 Department of Anesthesiology, Duke University School of Medicine, Durham, United States; 3 Departments of Anesthesiology and Surgery, Duke University School of Medicine, Durham, United States

**Keywords:** nutrition education, clinical nutrition, nutrition certification, medical school, continuing medical education, online nutrition fellowship, nutrition training, physician nutrition specialist, medical student, education, nutrition

## Abstract

The importance of nutrition in the development of disease, and in the recovery from illness, is among the most fundamental tenets in human biology and optimal health. Nutrition was fundamental in many traditional forms of medicine until its role in medical care experienced a rapid decline over the last century. We believe a key cause of the decline in nutrition's essential role in healthcare and preventative medicine is the escalating crisis of inadequate nutrition education in medical training. Recent data show 75% of United States medical schools have no required clinical nutrition classes and only 14% of residency programs have a required nutrition curriculum. More troubling, only 14% of current healthcare providers feel comfortable discussing nutrition with their patients. The purpose of this paper is to present the evidence illustrating the distinct lack of nutrition education in medical training. Further, we present key examples of existing formal nutrition curricula to incorporate nutritional science into all healthcare providers’ education and practices. We discuss existing nutrition fellowships and training programs, including the new Duke Online Clinical Nutrition Fellowship. We also cover a physician nutrition certification allowing physicians to pursue clinical nutrition as a career path. Finally, recent financial incentives and quality measures incentivizing healthcare provider nutrition education is discussed. Thus, in conclusion, we advocate the inclusion of nutrition education curricula as a priority in medical schools, graduate medical education, and continuing medical education. Formal clinical nutrition training should be a requirement for hospital leadership and administrators for all Parenteral Nutrition and Nutrition Team Physician Directors in hospitals worldwide, and this key clinical role must become an essential position in all hospitals. In addition, we immediately need to address the critical shortage of physician nutrition specialists who will serve as the next generation of leaders in clinical nutrition care and research.

**Statements of significance**

Currently, there is a crisis of inadequate nutrition education in medical training, with recent data showing 75% of United States medical schools and 86% of residency programs having no required clinical nutrition training; more troubling, only 14% of current healthcare providers feel comfortable discussing nutrition with their patients. Thus, we advocate for prioritizing nutrition education in medical schools, graduate medical education, and continuing education, as is being achieved by a growing number of formal nutrition curricula we describe here that aim to address the shortage of physician nutrition specialists needed to lead the future of clinical nutrition care and research.

## Introduction

“*I will apply dietetic and lifestyle measures to help the sick to my best ability and judgment; I will protect them from harm and injustice.*” The literal translation from Greek of the Hippocratic Oath.

*“Let food be thy medicine and medicine be thy food*” is often attributed to Hippocrates, but it is not clear he actually wrote these words [1]. However, his writings emphasize the essential role nutrition and lifestyle (above drugs and pharmacologic/surgical intervention) play in human health. This is clearly shown in the accurate and literal translation of the Hippocratic Oath above. Thus, the importance of nutrition in the development of disease, and in the recovery from illness, is among the most fundamental tenets in human biology and optimal health. Nutrition was fundamental in many traditional forms of medicine [2], until its role alongside curative medical therapy experienced a rapid decline in the last century [3]. We believe a key cause of the decline in nutrition's essential role in healthcare is the inadequate or complete lack of nutrition education in medical school, and especially post-graduate medical training. The challenge begins in medical school and graduate medical programs where the focus of nutrition education is often limited to the biochemical nature of nutritional deficiencies. This lack of nutrition training limits the impact that comprehensive medical care, inclusive of nutrition and lifestyle interventions, can have on the health and recovery of our patients and that of the community at large. This was recently emphasized in a JAMA opinion article on physician nutrition education [4], which highlighted why improving physician nutrition education must be an immediate priority with the following reasons:

1. A 2018 report from the United States Burden of Disease Collaborators highlighted poor diet quality as the top contributor to mortality in the United States [5]. If dietary habits do not improve, the incidence and costs associated with diet-related illnesses will escalate.
2. There is a growing emphasis on transitioning healthcare from managing diseases to promoting health and preventing illness, a shift that physicians will struggle to advance effectively without a strong grounding in clinical nutrition.
3. Patients are often overwhelmed by diet and health information from the media. Physicians must be well informed in nutrition to help patients navigate and make sense of often confusing and contradictory messages.
4. There is increasing focus on the wellbeing and self-care of healthcare providers and medical trainees. Clinical nutrition knowledge gained during training not only benefits patient care but can also enhance physicians’ personal health, making them more effective in advising others.

In this paper, we describe evidence for the “emergency” poor nutrition education in medical training has become. Even as medical schools have made recent and laudable efforts to equip their graduates with more robust nutrition curricula, these new doctors represent only a tiny fraction of the physician workforce, which comprises over 1 million doctors [6] who are already practicing in the United States. These physicians are already often pressed for time, given the average patient visit is ∼20 min [7], and most often do not have the training to effectively implement nutrition into their practice. We need other practical solutions for this group and thus we emphasize the need for comprehensive nutrition curricula in post-graduate medical education and, vitally, in continuing medical education. We then present key examples of existing formal post-graduate nutrition curricula that demonstrate new and unique opportunities to incorporate the application of evidence-based nutritional science into all healthcare providers’ practices. We also discuss an existing certification in nutrition for physicians and the vital need for the Physician Nutrition Specialist (PNS) and clinical nutrition as an essential specialty for physicians to consider as a career path. Finally, we discuss recent financial incentives and quality measures promoting improved nutrition care and the need for additional measures to incentivize nutrition education in medical training. Integration of nutrition education is essential to a complete medical education. However, there are currently inadequate hours spent on this topic in medical curricula at every level of training. This inadequacy has been well described. A survey of United States medical schools in 2010 reported that only 27% of schools met the minimum 25 required hours set by the National Academy of Sciences [8]. According to a 2018 survey, only 29% of United States medical schools met the recommended minimum 25 h of nutrition education, and only 14% of residency programs had a required nutrition curriculum [9,10]. Furthermore, these data show 75% of United States medical schools have no required specific clinical nutrition classes in their curricula [9]. More concerning, among subspecialists caring for diseases clearly impacted by nutrition, 90% reported receiving no or minimal nutrition education during fellowship training, and 59% reported no nutrition education during residency [10]. Similarly, based on a survey of faculty, fellows and medical residents at Langone Health-New York University, only 14% of healthcare providers felt comfortable discussing nutrition with their patients [11]. A recent 2019 systematic analysis of nutrition education worldwide publications examined data from 16 publications [12]. This analysis found that nutrition is poorly incorporated into medical education, regardless of country, setting, or year of medical education. The reviewed studies consistently show that medical students want to receive nutrition education to develop expertise in nutrition therapy and optimal care. This analysis further found medical students’ perceptions on receiving inadequate nutrition education are consistent with all existing literature [8,13]. Unfortunately, data showed that these common nutrition knowledge deficits continued well into their future medical practice [8,14]. Finally, medical directors [15] and faculty similarly agree that the nutrition education provided to medical students is inadequate and insufficient [16]. In 1985, the United States National Research Council’s Committee on Nutrition in Medical Education published a review of the current state of nutrition education in United States medical schools and set recommendations to improve adequacy of training [17]. They identified several primary barriers to effective implementation of nutrition programs, including the low number of Physician Nutrition Specialists who could serve as champions and a lack of up-to-date nutrition practices among active practitioners who would serve as role models. Underscoring the relevance and timeliness of the issue, in 2023 the Accreditation Council for Graduate Medical Education (ACGME) developed guidance for educators on improving education in undergraduate and graduate medical education. The summit recommendations included the development of an integrated, longitudinal, and interprofessional nutrition curriculum [18]. Without adequate nutrition education, it is clear that physicians are not prepared or able to provide the highest quality care to patients. Nutrition education deficiency undoubtedly affects physicians’ knowledge, skills, and confidence to implement nutrition therapy and counseling into patient care.

Nutrition therapy in illness is often complex and nuanced, with multiple factors influencing a patient's nutritional status and requirements. This makes it even more challenging for healthcare providers with little-to-no nutrition training to be confident in their abilities to assess nutritional status, provide evidence-based nutrition counseling or even make appropriate referrals to PNSs and registered dietitian nutritionists (RDNs). Overall, nutrition education is often inadequate in medical school and residency training, leaving healthcare professionals ill-equipped to address the complex nutritional needs of their patients. It is also important to acknowledge that the breadth and depth of nutrition education RDNs receive over 4 y cannot be expected to be covered by all physicians. Certainly, an important aspect of nutrition education must be recognizing the crucial roles of RDNs and facilitating collaboration with a multidisciplinary team that includes RDNs in managing the range of disease states physicians are routinely asked to provide care for. As described in this manuscript, and is well known to established practitioners in the field, physician specialists in nutrition need to work collaboratively with RDNs, and draw upon their vast training and clinical experience.

Medical schools are attempting to address this deficiency in nutrition education. Paralleling the rise of the “food as medicine” and “culinary medicine” paradigms, an increasing number of United States medical schools support produce prescription programs and patient-facing education sessions on medically tailored meal preparation and grocery shopping – often as student-run initiatives or in conjunction with student-run free clinics [19]. These offer students interested in medical nutrition opportunities to supplement their formal curricula with nutrition-centered, experiential service learning. As community-facing efforts that generally target marginalized populations, such programs also provide valuable opportunities for students to learn about nutrition through a public health lens by allowing them to tangibly interact with and address food insecurity, nutrition insecurity, and education as social drivers of health. However, they may not address skills essential to nutritional assessment and management in hospitalized patients, such as competency in conducting nutrition-focused histories and physical examinations, addressing physical and cognitive barriers to adequate caloric intake, and prescribing nutrition therapy.

Several United States medical schools are already taking steps to address this issue by creating nutrition-focused electives and rotations or integrating nutrition into existing courses [20]. The University of California, San Francisco School of Medicine, the University of North Carolina at Chapel Hill School of Medicine, the Geisel School of Medicine at Dartmouth, and the Tulane University School of Medicine are examples of institutions that have integrated nutrition throughout their curriculum. A recent review described results of 30 published, nutrition-focused education experiences in United States medical schools [20]. These were described as integrated courses or curricula (*n* = 10, 33%), sessions (*n* = 17, 57%), or nutrition electives (*n* = 3, 10%) that were available to students. The review described considerable heterogeneity in assessment and teaching methods used. The most common teaching modality was lectures (*n* = 21, 70%), with learning most often assessed via pre- and/or postsurveys (*n* = 19, 79%). Six of the studies (26%) reported on outcomes after completion of nutrition education experiences via objective measures, such as examinations or standardized patient experience scores. This important review revealed that minimal and inconsistent data exist on medical school nutrition learning experiences [20] and that this area needs further research and description [20]. However, it is encouraging that an increasing number of schools are attempting to add nutrition curricula to medical school training.

## The Urgent Need to Address Postgraduate and Continuing Medical Education Nutrition Training

Although medical schools try to address the deficiency of nutrition training at the medical student level, there are currently ∼1 million practicing physicians in the United States and more worldwide for whom we must, as an immediate priority, develop easily accessible continuing medical education programs to improve nutrition care. Further, there is a rapidly growing shortage in PNSs who focus on clinical nutrition as a significant part of their medical practice worldwide [21]. Not surprisingly, physician membership in leading professional nutrition societies has been decreasing over the past 20–30 y. The number of physicians in the American Society for Parenteral and Enteral Nutrition in 2009 was barely one-third the number seen in 1990 (down to <13% of the total membership) [21]. Recently, the number of physicians in the American Society for Nutrition fell to a range of between 100 and 150 members [21]. The number of physicians sitting for board examinations in nutrition also decreased, such that over the past 4 y only between 27 and 31 physicians have sat for 1 of 3 examinations in clinical nutrition [21]. Expert panels have been convened to address this pressing shortage, including the “Summit on Increasing Physician Nutrition Experts.” [21,22].This Summit strongly recommended that development of dedicated continuous Nutrition and Health Promotion (NOHP) fellowships is urgently needed. This has been validated by other expert groups, who have provided recommendations for improving medical nutrition education and practice and at the residency and fellowship training level [23].

Several residency programs have taken up the mantle of incorporating nutrition into their training. For example, the Anesthesiology Residency Program at Duke University Medical Center has a dedicated nutrition curriculum that includes didactic sessions, case-based discussions, and hands-on experiences. The program aims to provide residents with the knowledge and skills necessary to recognize and address the nutritional needs of their patients. Similarly, the University of Wisconsin Anesthesiology Residency Program has a nutrition curriculum covering preoperative optimization, parenteral and enteral nutrition support, and managing nutrition-related complications. In addition, programs such as Stanford Medicine’s “*Introduction to Food and Health*” continuing medical education (CME) course have been started with excellent success. Physicians completing this course reported being far more likely to discuss nutrition with their patients in their practice [24]. Additionally, the *American College of Lifestyle Medicine* offers certification programs that include comprehensive nutrition education for clinicians. These programs focus on the practical application of nutrition science in chronic disease prevention and management. Further, clinical nutrition societies, such as the European Society for Parenteral and Enteral Nutrition (ESPEN) and the American Society for Parenteral and Enteral Nutrition (ASPEN) have created a range of virtual and in-person courses and webinars for nutrition professionals to further their nutrition knowledge. These include the ESPEN Life-Long Learning (LLL) program (website: https://www.espen.org/education/lll-programme). The LLL Programme in Clinical Nutrition and Metabolism is based on an Educational Curriculum of >120 training modules, created and peer-reviewed by recognized experts in the field. It offers online training and live courses using a variety of modern training methodologies and resources. The main educational unit of the LLL programme is the module. Each module includes an updated review of a certain problem, a clinical case, a self-assessment test, and a grading quiz. Modules on related subject areas are grouped into 40 topics, making it easier for users to select modules in a particular field of interest. The American Society of Nutrition (ASN) also has a Physician Group Engaging Members Community to further physician nutrition education. Although these are well taught on a range of topics, they may not always be targeted or promoted to physicians and providers without significant previous nutrition training.

Furthermore, the Nestlé Nutrition Institute Clinical Nutrition Fellowship for Physicians is an in-person program that has been established to foster nutrition knowledge in the care of acute and chronically ill patients (see: https://www.nestlemedicalhub.com/program-information/nestle-nutrition-institute-clinical-nutrition-fellowship-for-physicians). This program aims to train licensed medical or surgical physicians in specialized medical nutrition. The program accepts a limited number of physicians each year via a competitive application process. The program has traditionally consisted of high-level, in-person experiences for multiple weeks at several institutions where key program faculty are located across the United States. This has been an excellent resource for highly committed United States medical or surgical physicians. One potential limitation to the program for practicing clinicians is that, although an excellent training experience, the program requires participants to travel to and live for multiple weeks at 2 United States host institutions. This is not always practical for physicians already in practice. This also can pose a significant challenge for clinicians from outside the United States, especially in resource-limited developing nations.

**FIGURE 1:**
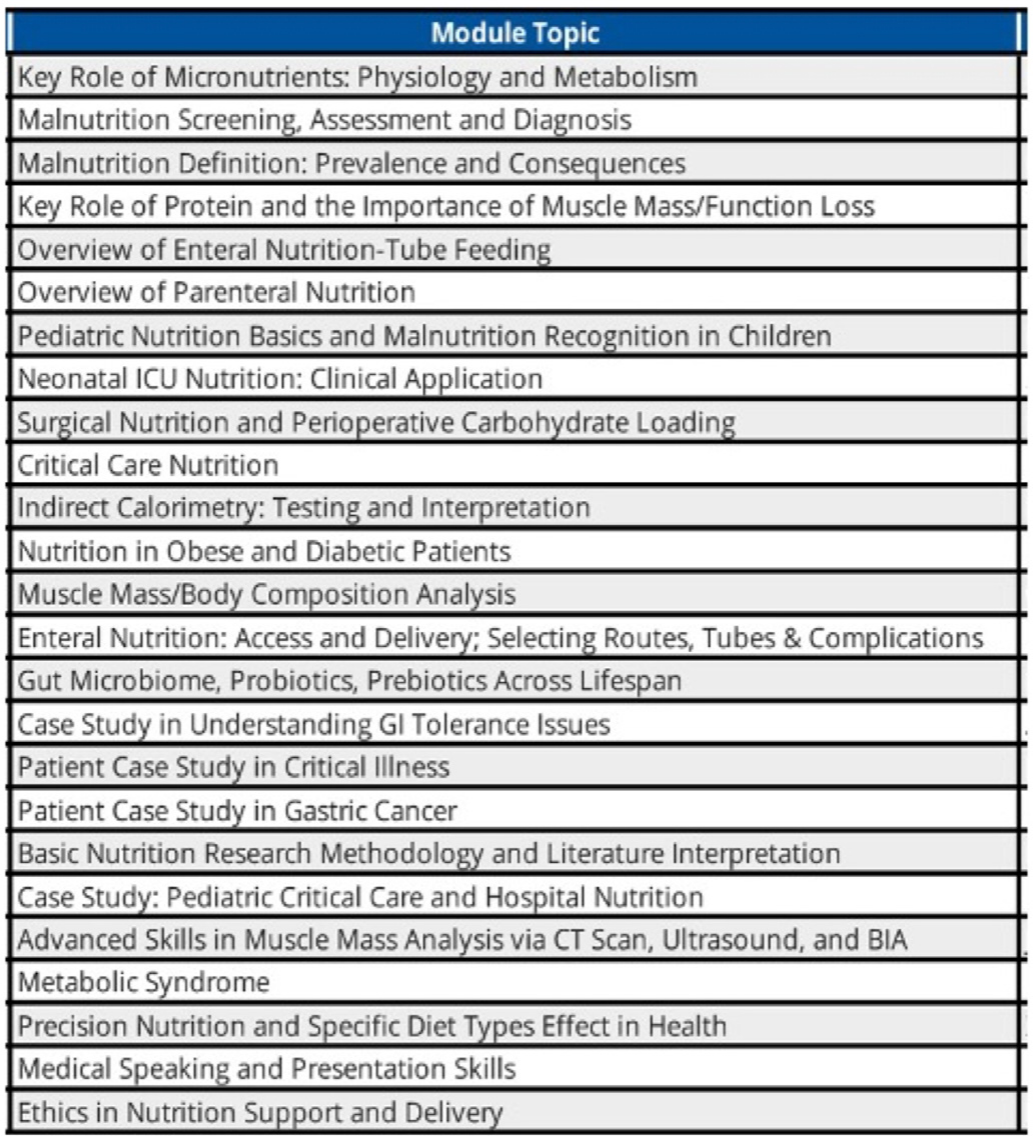
Representative course list for Duke Online Clinical Nutrition Online Fellowship (from 2023 Online Fellowship year).

To further augment existing opportunities for post-graduate nutrition training, Duke University has started the Duke Online Clinical Nutrition Fellowship, which provides online, fellowship-level education in clinical nutrition available to physicians, advanced practice providers (nurse practitioners [NPs] or physicians assistants [PAs]), RDNs, and pharmacists. Topics and settings included in this program include nutrition assessment and therapy in a wide variety of critical care and perioperative settings, which can be taken as stand-alone pieces or as part of the full program (see Figure 1 for a representative list). The program aims to improve clinical nutrition education among physicians and other healthcare providers worldwide (for more information see: https://anesthesiology.duke.edu/clinical-nutrition-course). Continuing medical education credits can also be gained, providing further incentives to current practitioners. The program also provides tuition discounts and scholarships to physicians from underserved nations and to RDNs worldwide. The Duke Online Nutrition Fellowship utilizes unrestricted educational grant funding from Abbott Nutrition Inc. to make these reduced costs possible.

In the first 2 y of the program, the Duke Online Clinical Nutrition Fellowship has enrolled over a hundred participants from 15 countries around the world. Of these, 9 physicians from the United States, Latin America, Australia, and Asia have completed all the interactive modules. These physicians come from a variety of specialties including surgery, internal medicine, hospital medicine, and critical care medicine. Similarly, the 2024 program drew diverse international participation from physicians and RDNs from 13 countries and 4 continents. Twenty-four physicians and 12 RDs graduated from the Fellowship in 2024. As described below, this training also serves as the mentored training requirement to sit for the National Board of Physician Nutrition Specialists (NBPNS) examination, and recent Duke Clinical Nutrition Fellowship graduates have successfully taken and passed the NBPNS examination, thus attaining clinical nutrition board certification.

Each module is 2 wk long, includes prerecorded material for participants to review and is followed by moderated online discussions. A key goal of these interactive modules is to grow relationships and collaborations between attendees and international faculty experts, which could help guide future education, clinical care, and research. Because the participants are active practitioners, the discussions are often focused on practical matters, such as methods to overcome barriers, and pathways for the implementation of current science. Key features of the program are discussion board and live-video–based interactive sessions online, where faculty moderate interaction among participants to promote multidisciplinary learning. To reflect this ethos, 5 of the 22 faculty in the 2023 program were RDs. These features will help build key relationships to drive the creation of the next generation of physician nutrition specialists and leaders.

Currently, the Duke Online Clinical Nutrition Fellowship also offers a focused Research Track where physician fellows and RDN full-course participants will have the opportunity to be trained in clinical nutrition research methodology including (1) clinical research design and conduct, (2) database analysis, (3) statistical design and analysis, (4) quality improvement project (QIP) development/analysis, (5) abstract/manuscript writing skills, (6) grantsmanship for future projects, and (7) presentation skills for oral presentation of research projects. The research track is targeted to assist in creation of future academic leaders in clinical nutrition. As such, participants will work with program faculty and epidemiologists/statisticians on large database analysis of nutrition-related questions. This will lead to (1) abstracts or abstracts submitted and presented at ASPEN, ESPEN, and/or key regional PEN society meetings, (2) presentation of projects at key ASPEN, ESPEN, and/or key regional PEN society meetings, (3) writing of a manuscript with a “Research Fellow” as first author and key Online Clinical Nutrition Fellowship faculty as co-authors to help guide and instruct the “Research Fellows” in academic writing, journal selection, and publication. Finally, the Duke Online Clinical Nutrition Fellowship is developing a proposed in-person clinical nutrition training experience or preceptorship that will occur at Duke University School of Medicine/Hospitals. This will include direct interaction with faculty and staff on clinical rounds. Key training on new clinical nutrition technologies including indirect calorimetry, muscle ultrasound, computed tomography scan–based muscle assessment, bioimpedance analysis (BIA), and cardiopulmonary exercise testing (CPET) will also be included. In addition, potential for in-person research experiences and training will be considered for interested trainees. We feel that committed clinical nutrition training programs like the Duke Online Clinical Nutrition Fellowship are needed to address the well-described crucial shortage of PNSs worldwide [21].

## Certification in Nutrition for Physicians

There is a need for PNSs who can provide leadership about the vital relationship between diet and disease to both healthcare teams and the lay public. The NBPNS maintains credentialing standards for assessment and certification of physicians seeking recognition as a PNS (see nbpns.org). Certification as a Diplomate of the NBPNS signifies a physician as having achieved a high level of competency and understanding in scientifically sound, evidenced-based medical nutrition.

PNSs help individual patients, the community at large, and hospitals (in both outpatient and inpatient settings) incorporate best practices for nutrition therapy in the prevention and treatment of disease. Vitally, they also lead referrals to multidisciplinary teams that include RDNs, social workers, culinary experts, and psychologists [25].

PNSs are recognized not only for their nutrition expertise but also for their skills in leading nutrition-focused multidisciplinary care teams, teaching best nutrition practices in academic health centers and medical schools, and integrating evidence-based medical nutrition therapy in clinical practice. PNSs champion the incorporation of scientifically rigorous research into nutritional approaches for preventing and treating disease in both the inpatient and outpatient settings. Although PNSs have diverse backgrounds and training in numerous medical specialties, including, but not limited to, internal medicine, surgery, gastroenterology, critical care, and endocrinology, all are recognized by their peers as experts and leaders of nutrition in their field of practice.

To be eligible to take the NBPNS examination, practitioners must demonstrate expertise in nutrition by a few means. Possible pathways include mentored training in clinical nutrition, dedicated service on a hospital multidisciplinary nutrition team, publication of nutrition research, and various other demonstrations of local or regional leadership in nutrition. The Duke Online Clinical Nutrition Fellowship provides one of the many pathways for mentored training, which is particularly useful for those who do not have such training available at their own institutions.

## Need for Nutrition-Focused Financial Incentives and Quality Measures to Drive Increased Nutrition Education in Healthcare

Despite a strong understanding of nutrition knowledge and training, physicians and healthcare providers also lack the financial drivers necessary to prioritize optimal nutrition therapy in their daily practice or to incentivize pursuit of a Clinical Nutrition Specialist certification. For example, in perioperative and surgical care it is well known that nutrition optimization following a perioperative malnutrition diagnosis reduces mortality and improves outcomes from major surgery. However, in the United States, anesthesiologists’ and surgeons’ reimbursement is currently based on time in the operative room or relative value units (RVUs) [26,27]. As a result, although surgeons and anesthesiologists are motivated to improve patient outcomes, they currently do not work in a system that allows appropriate time in the preoperative (or postoperative) period to assess and optimize a patient's nutritional needs and status. This is an enormous, missed opportunity to improve patient outcomes and reduce costs, as studies consistently show that nutrition plays an essential role in complication rates, mortality, and patient recovery [[28], [29], [30]]. Thus, a compelling need exists to align financial incentives for optimal nutrition care with opportunities to improve patient outcomes. Our hope is that improved billing, leading to better reimbursement, would in turn incentivize healthcare systems to allocate time, personnel, and resources to the diagnosis and treatment of malnutrition. At the federal level, the Congressional introduction of the Medical Nutrition Therapy (MNT) Act of 2023 represents a positive step toward broadening reimbursement for nutritional services. Historically, a referral from a physician has been required for a Medicare beneficiary to utilize MNT services under Medicare Part B, but this bill would additionally authorize nurse practitioners, physician assistants, clinical nurse specialists, and psychologists to refer patients experiencing diet-related chronic illnesses (prediabetes, obesity, hypertension, dyslipidemia, malnutrition, etc.) for outpatient MNT as well. Although this speaks to the growing recognition that the physician workforce should work in concert with other professions to address nutrition-related problems, it also underscores the need for similar reimbursement models and practices in the inpatient setting. In general, payor incentives also need to change from the current, largely “sick-care” model, to preventative interventions.

Further, a recent large multicenter trial showed that the basic ability of a healthcare provider to rapidly recognize and treat malnutrition upon hospital admission via the use of a simple screening pathway and inexpensive oral nutrition supplement (ONS) drinks led to significant reductions in mortality and complications at 30 d, as well as a significant improvement in recovery of functional independence (*P* < 0.006) and EQ-5D quality of life at 30 d (*P* = 0.018) [31]. The screening was performed by a specialist dietician, which further emphasizes the collaborative, multidisciplinary approach necessary to tackle malnutrition. This seminal study also demonstrated that basic healthcare provider recognition of malnutrition at hospital admission and provision of early in-hospital nutritional support for medical inpatients was a highly cost-effective intervention to reduce risks for intensive care unit admissions and hospital-associated complications, while improving patient survival. The positive clinical and economic benefits of malnutrition recognition and nutritional therapy in at-risk medical inpatients calls for comprehensive nutrition education programs that emphasize the need for healthcare provider training in malnutrition screening, consultation, and nutritional therapy [32].

A current incentive for healthcare providers to seek increased initial and continuing education in nutrition care is that a malnutrition diagnosis, when properly made using ASPEN, Academy of Nutrition and Dietetics or Global Leadership Initiative on Malnutrition (GLIM) criteria, can modify a patient’s predicted severity of illness, predicted mortality risk, and, importantly, a hospital’s overall performance index. It is essential that healthcare providers are informed that a properly coded malnutrition diagnosis is a well-recognized major complication code (MSS) that increases complexity of care and is a diagnosis-related group modifier of reimbursement. Thus, malnutrition as a major complication or comorbidity (MCC) can significantly increase hospital reimbursement. It also modifies the observed-to-expected mortality ratios, which are directly associated with hospital quality measures and national hospital rankings (i.e., United States News and World Report rankings of hospitals). Unfortunately, although it is well known that >1 in every 3 hospitalized patients is malnourished by diagnostic criteria upon hospital admission [33], recent 2018 Healthcare Cost and Utilization Project (HCUP) data estimate that only 8.9% of malnourished patients are being recognized, diagnosed, and ultimately coded [34]. This represents a major unrecognized opportunity for improved malnutrition diagnosis and subsequent increased reimbursement if simple improvements in medical provider nutrition education were to be implemented.

In an effort to improve health care quality, CMS established the Hospital Inpatient Quality Reporting (IQR) Program in 2003. As part of the IQR, CMS develops quality measures called electronic clinical quality measures (eCQMs), which are tracked via data from the hospital’s electronic health record (EHR). Hospitals are required to track 3 mandatory measures, as well as 3 self-selected measures from a list of 10. Performance is reported publicly, and participation affects Medicare payments and hospital reimbursement. Hospitals that participate in the Hospital IQR program receive an increase in payment for Medicare patients’ hospital stays, whereas hospitals that do not meet the minimum reporting requirements receive a decrease in payment [35]. These financial consequences are meant to incentivize hospitals to provide quality, evidence-based care, which for the first time now includes optimal malnutrition diagnosis and nutrition care. This is a major advance in the prioritization of good nutrition care as a primary mission of hospital and healthcare quality and practice.

**FIGURE 2:**
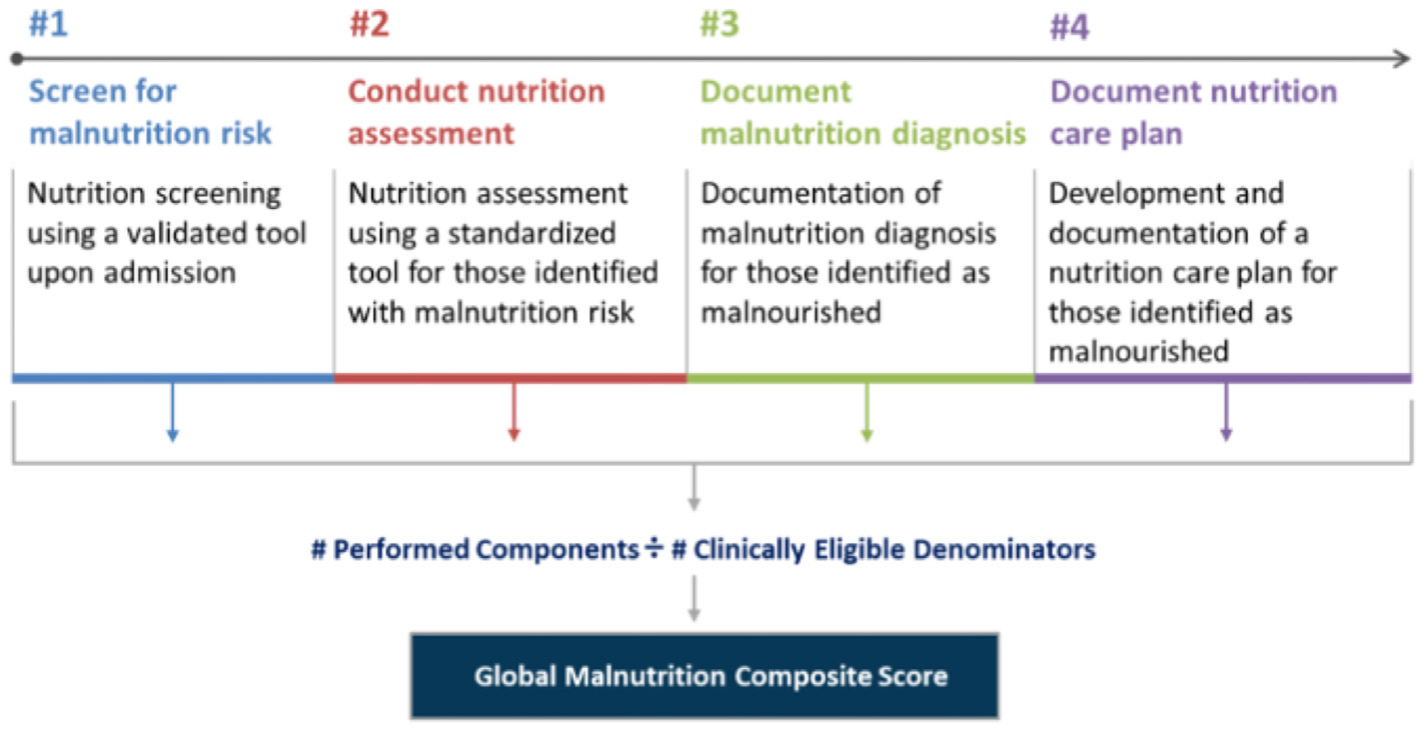
Four essential components of Global Malnutrition Composite Score. As a new CMS quality measure, the GMCS is the first nutrition-focused quality measure in any CMS payment program. It is also the first electronically specified composite measure. It addresses malnutrition for hospitalized adults via 4 essential components reflecting key steps in the inpatient malnutrition care workflow necessary to identify and manage malnutrition risk in a timely and effective manner. (Adapted from MQii Malnutrition Quality Improvement Website https://malnutritionquality.org/gmcs-for-iqr/). CMS, Centers for Medicare and Medicaid Services; GMCS, Global Malnutrition Composite Score.

Finally, a major achievement in advancing the role of optimal nutrition care and highlighting the pressing need for increased nutrition education in medical training was the recent approval by the Centers for Medicare & Medicaid Services (CMS) of the Global Malnutrition Composite Score (GMCS). The GMCS is a new quality measure that will be available for reporting in 2024 by hospitals participating in the CMS Hospital IQR Program. Stewarded by the Academy of Nutrition and Dietetics, the GMCS is the first nutrition-focused quality measure in any CMS payment program. It is also the first electronically specified composite measure. It addresses malnutrition for hospitalized adults via 4 essential components (see Figure 2), reflecting the key steps in the inpatient malnutrition care workflow that are necessary to identify and manage malnutrition risk in a timely and effective manner [35].

In conclusion, although it is widely acknowledged that nutrition education needs to be urgently instituted in medical schools and graduate medical education, there is a simultaneous need to help active practitioners improve their practice of clinical nutrition. Thus, we advocate that comprehensive nutrition education curricula need to be immediately instituted in medical schools, graduate medical education, and continuing medical education. This clinical nutrition training needs to be made available to physicians and healthcare providers worldwide, regardless of location, and account for often-limited financial resources in developing nations. We advocate that formal clinical nutrition training should be required by hospital leaders and administrators for all Parenteral Nutrition or Nutrition Team Physician Directors in hospitals worldwide. Further, it is essential that all hospitals be required to invest in a TPN and/or Clinical Nutrition Physician to create nutrition “champions” to work in concert with and support the work of RDs and other nutrition providers in their essential roles. In addition, we desperately need to address the critical shortage in PNSs who will serve as the next generation of leaders in clinical nutrition care and research.

## Author contributions

The authors’ responsibilities were as follows – PEW, SK: designed and conceived the manuscript; PEW, TS, SK: conducted background research for the manuscript; PEW, SK, TS: wrote the paper; PEW: had primary responsibility for final content; and all authors read and approved the final manuscript.

## Conflicts of interest

Paul E. Wischmeyer reports receiving investigator-initiated grant funding related to this work from the National Institutes of Health, Department of Defense, Abbott, Baxter, and Fresenius. Wischmeyer has served as a consultant to Abbott, Fresenius, Baxter, Mend Inc., and Nutricia for research related to this work. Wischmeyer received unrestricted gift donations for nutrition research from Musclesound and DSM. Wischmeyer has received honoraria or travel expenses for CME lectures on improving nutrition care from Abbott, Baxter, Fresenius, Danone-Nutricia, DSM, and Nestle. SK and TS: Report no conflicts of interest.

## Funding

No outside support was obtained. Support was provided solely from institutional and/or departmental sources.
